# Emerging roles of long non-coding RNAs in keloids

**DOI:** 10.3389/fcell.2022.963524

**Published:** 2022-08-15

**Authors:** Xin Yu, Xueqing Zhu, Hongjun Xu, Linfeng Li

**Affiliations:** Department of Dermatology, Beijing Friendship Hospital, Capital Medical University, Beijing, China

**Keywords:** long non-coding RNAs, keloids, HOXA11-AS, H19, Smad5 protein

## Abstract

Keloids are pathologic wound healing conditions caused by fibroblast hyperproliferation and excess collagen deposition following skin injury or irritation, which significantly impact patients by causing psychosocial and functional distress. Extracellular matrix (ECM) deposition and human fibroblast proliferation represents the main pathophysiology of keloid. Long non-coding RNAs (LncRNAs) play important roles in many biological and pathological processes, including development, differentiation and carcinogenesis. Recently, accumulating evidences have demonstrated that deregulated lncRNAs contribute to keloids formation. The present review summarizes the researches of deregulated lncRNAs in keloid. Exploring lncRNA-based methods hold promise as new effective therapies against keloid.

## Introduction

Keloids are pathological scars characterized by firm, raised, erythematous plaques or nodules following cutaneous injury, which under normal conditions results in wound healing with a flat scar (Wolfram et al., 2009). Keloids grow abnormally beyond wound margin and may appear several years after cutaneous injury (Atiyeh et al., 2005). Historically, keloids are characterized by a thick dermis with infiltrated inflammatory cells and marked collagen deposition (Gauglitz et al., 2011; Ghazawi et al., 2018a). Keloids are pathological scarring conditions and cause various problems for patients, including pruritus, pain, functional impairment, cosmetic distortion, and psychological distress. Fibroblast proliferation and extracellular matrix (ECM) deposition represents the main pathophysiology of keloids (Ghazawi et al., 2018b). The ECM components of keloid consist of many components, including collagen 1 (Col1), collagen 3 (Col3), α-smooth muscle actin (α-SMA) and fibronectin (FN) (Sidgwick and Bayat, 2012; Xue and Jackson, 2015). Overabundance of ECM is caused by hyperproliferation of human keloid fibroblasts (HKFs). Current treatments for keloids, which include surgical excision, intralesional corticosteroid injections, cryotherapy, pressure therapy, radiotherapy and laser therapy, are not satisfactory (Arno et al., 2014; Kaartinen, 2016). Therefore, elucidating the underlying mechanism is crucial to explore new therapeutic targets of keloid.

Recent advances have proved that most human genome is transcribed to RNAs that lack the capacity to encode proteins (non-coding RNAs). Long non-coding RNAs (LncRNAs), are defined as transcripts longer than 200 nucleotides in length without protein-coding capacity (Jarroux et al., 2017). Increasing studies have demonstrated that lncRNAs play important role in regulating gene expression in many biological and pathological processes, including development, differentiation and carcinogenesis (Quinn and Chang, 2016; Yu et al., 2018). LncRNAs can activate or inhibit gene expression through several mechanisms. For instance, lncRNAs can form riboprotein complexes through interacting with proteins, which regulate protein function and localization (Wilusz et al., 2009). Recently, emerging evidence have suggested that deregulated lncRNAs play significant roles in keloid formation by regulating many processes, including fibroblast proliferation and extracellular matrix (ECM) deposition. Therefore, lncRNAs may serve as potential diagnostic and therapeutic biomarkers in keloids.

### Long non-coding RNAs in Keloid

Liang et al. found that more than 2,500 lncRNAs were differentially expressed between keloid tissue and the normal human skin by microarray analysis and qRT-PCR. Among them, 1,731 lncRNAs was upregulated and 782 lncRNAs was downregulated (Liang et al., 2015). Further bioinformation analysis revealeld that lncRNA CACNA1G-AS1 (CAS1), highly expressed in keloid tissue, may act crucial role in keloid formation. Another study identified a total of 2068 lncRNAs were differentially expressed in earlobe keloid compared with normal tissues using microarray analysis. Among them, expression of 1290 lncRNAs was upregulated, and 778 lncRNAs downregulated (Guo et al., 2016).

Another study identified a total of 319 keloid-specific lncRNAs using RNA-seq and miRNA-seq (Duan et al., 2020). The study also identified two competing endogenous RNAs (ceRNA) network of mRNA/miRNA/lncRNA in the regulation of the actin cytokeleton pathway. LncRNA GLB1L-1 was a ceRNA which decreased EGFR expression via sponging miR-370-3p. LncRNA CASP9-3 was a ceRNA which increased ITGB5 expression via sponging miR-204. This laid a foundation for future investigations of pathogenesis and therapeutic targets for keloid.

To identify lncRNAs differentially expressed during keloid formation, secondary analysis of keloid-related sequencing and microarray data were downloaded from the open-source Gene Expression Omnibus (GEO) database. A total of 2356 lncRNAs was changed before and after injury in keloid-prone groups (Deng et al., 2020). Among these deregulated lncRNAs, 1306 lncRNAs were increased and 1050 lncRNAs were decreased in keloid-prone groups after injury. However, 2547 differential expressed lncRNAs—1479 increased and 1068 decreased lncRNAs were identified in the control group after injury. Furthermore, 6 lncRNAs, namely, 2 upregulated (DLEU2 and AP000317.2) and 4 downregulated (ADIRF-AS1, AC006333.2, AL137127.1 and LINC01725) lncRNAs, were expressed only in the keloid-prone group and were used to construct a ceRNA network. Moreover, the expression of DLEU2 and ADIRF-AS1 were significantly different in fibroblasts in keloid scars compared with the normal skin. However, the expression of AC006333.2, AL137127.1, and LINC01725 was not statistically significant between two groups. Specifically, many studies have showed that DLEU2 was frequently deleted or epigenetically suppressed in leukaemia and acted as a tumour suppressor (Zhou et al., 2019a; Wu et al., 2020). Therefore, DLEU2 was predicted to play an important role in keloid formation and wound healing through regulating fibroblast proliferation, differentiation, and apoptosis. However, more basic and clinical experimental data are needed to verify this hypothesis.

A recent study identified 11 differentially expressed 11 EMT-related lncRNAs and 16 mRNA in keloid tissues versus normal tissues (Chen et al., 2022). These 16 differentially expressed mRNAs played key roles in the extracellular matrix, cellular processes, protein binding, the Set1C/COMPASS complex and histone acetyltransferase activity, as well as in pathways involved in malignancies. Morever, lncRNA XLOC_000587 may increase cell proliferation and migration by enhancing the expression of ENAH, while AF268386 may facilitate the invasive growth of keloids by upregulating DDR2 (Table 1).

**TABLE 1:** LncRNAs expression profiles in Keloids.

Num	Method	sample	upregulated	downregulated	Reference
1	Microarray RT-PCR	keloid	1,731 lncRNAs	782 lncRNAs	14
2	Microarray RT-PCR	keloid	1290 lncRNAs	778 lncRNAs	15
3	Microarray RT-PCR	keloid	251 lncRNAs	68 lncRNAs	16
4	RNA sequencing RT-PCR	Injured keloid	1306 lncRNAs	1050 lncRNAs	17
5	RNA sequencing RT-PCR	keloid	7 EMT-lncRNAs	4 EMT-lncRNAs	20

### Long non-coding RNAs act as miRNA sponges in regulating human keloid fibroblasts proliferation, apoptosis, cell cycle, tumor growth, epithelial-mesenchymal transition, migration, invasion and metastasis

The development of keloid was significantly related with HKFs proliferation, migration, invasion, and apoptosis. LncRNAs and miRNAs can communicate with and co-regulate each other (Table 1). These lncRNAs, known as competing endogenous RNAs (ceRNAs), negatively regulate miRNAs by acting as miRNA sponges [23]. LncRNAs contain similar miRNA target sequences and served as “sponges’”, correspondingly regulating miRNA activity. LncRNAs could prevent miRNAs from acting on mRNAs and contributed to enhanced translations of their target mRNAs (Zhao et al., 2020a).(Table 2)

**TABLE 2:** Deregulated LncRNAs in keloids.

LncRNA	Expression	Function	Target	Reference
H19	Upregulated in human keloid and HKFs	Promotes HKFs proliferation	miR‐29a, miR-214-5p, miR-769-5p, miR-196b-5p	27-32
HOXA11-AS	Upregulated in human keloid and HKFs	Promotes HKFs proliferation and migration	miR-124-3p, miR-205-5p, miR-148b-3p	33-36,38
CAS1	Upregulated in human keloid and HKFs	Promotes HKFs proliferation, invasion and migration while suppresses apoptosis	miR-205	39,40
LINC01116	Upregulated in human keloid and HKFs	Promotes HKFs proliferation, invasion, migration, and ECM production while inhibited HKFs apoptosis	miR-203, miR-3141	42,43
LncRNA-ATB	Upregulated in human keloid and HKFs	Promotes the autocrine secretion of TGF-β2 in KF	miR-200c, ZNF217	46,47
AC073257.2	Upregulated in human keloid	Promotes HKFs growth and proliferation	GLI2	48
LINC00937	Downregulated in human keloid and HKFs	Inhibits ECM deposition and HKFs proliferation	miR-28-5p	49

CAS1, CACNA1G-AS1; HKFs, human keloid fibroblasts; ECM, extracellular matrix; HOXA11-AS, homeobox (HOX) A11 antisense; CACNA1G-AS1, calcium voltage-gated channel subunit alpha1 G antisense RNA, 1; LncRNA-ATB, LncRNA-activated by TGF-β (lncRNA-ATB).

### H19

**FIGURE 1:**
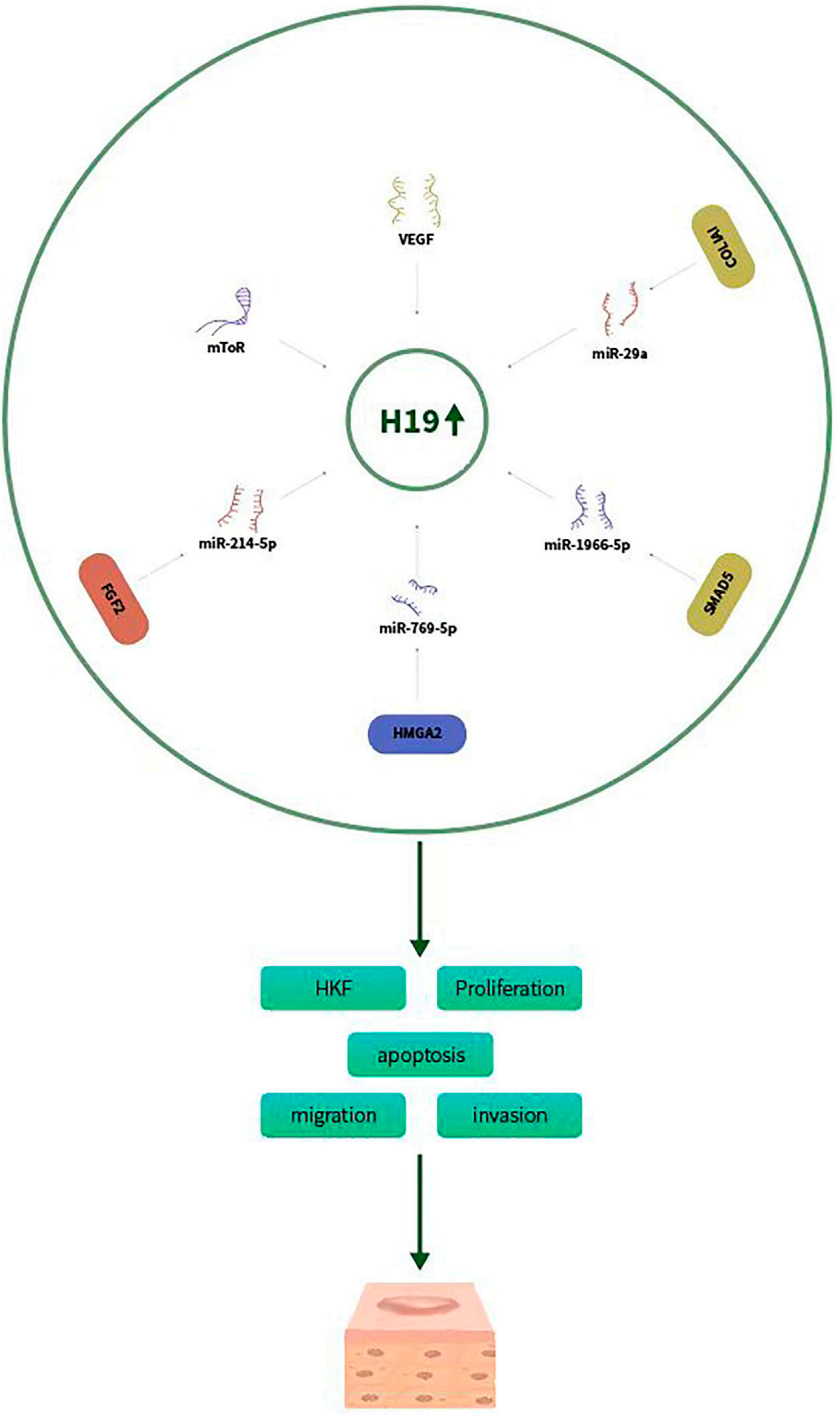
H19 promotes keloid progression via sponging miR-29a, miR-214-5p/FGF2, miR-769-5p/EIF3A, and miR-196b-5p/SMAD5 aixs.

LncRNA-H19 was one of the first reported lncRNAs and associated with proliferation in many tumors (Yoshimura et al., 2018). H19 can promote the development and progression of many malignancies, including lung cancer, hepatocellular carcinoma and nasopharyngeal carcinoma (Zhou et al., 2019b; Ye et al., 2019; Zhang et al., 2019; Zhao et al., 2019). H19 play a crucial role in the development of human fibrotic diseases, such as pulmonary fibrosis, liver fibrosis, myocardial and fibrosis renal fibrosis (Li et al., 2020). H19 expression was upregulated in human keloids compared with normal scars and normal skin controls. H19 regulates the proliferation of HKF. Previous study had proved that the mammalian target of rapamycin (mTOR) signaling pathway contributes to the development of keloid (Ong et al., 2007). Knockdown of H19 decreased the expression of mTOR and vascular endothelial growth factor (VEGF), thus suppressing HKFs proliferation (Zhang et al., 2016). H19 markedly promoted HKFs proliferation and metastasis by targeting miR‐29a. Moreover, collagen type I alpha 1 (COL1A1), a vital gene involved in keloid scarring, was increased by H19 and yet inhibited by miR‐29a (Wang et al., 2020). Lu et al. also demonstrated that H19 promoted keloid development by targeting miR-214-5p/FGF2 axis (Lu et al., 2021). H19 was overexpressed in KD tissues and HKFs compared with normal skin controls and normal fibroblasts, respectively. Silencing of H19 inhibited glycolysis, migration and invasion of HKFs exposed to hypoxia, reversed by downregulation of miR-214-5p or upregulation of FGF2. In summary, H19 might be a potential marker of keloid diagnosis or treatment. A recent study also showed that H19 expression was markedly increased in keloid tissues and HKFs. H19 knockdown decreased cell proliferation, migration, invasion, ECM accumulation, but accelerated apoptosis of HKFs (Xu et al., 2021). In addition, H19 was proved to sponge miR-769-5p and its inhibition overturned the effects of H19 knockdown. Moreover, eukaryotic initiation factor 3A (EIF3A) was a target of miR-769-5p, and its overexpression inverted the effect of miR-769-5p on keloid formation. In summary, H19 might may be an active mode in keloid formation and serve as a potential therapeutic target for keloid patients. Verification of the molecular and cellular mechanisms of H19 in fibrotic diseases may contribute to the development of novel therapeutic approaches for fibrotic diseases including keloid. H19 expression was increased in keloid tissue and fibroblasts, whereas miR-196b-5p expression was decreased. Overexpression of H19 or SMAD5 and knockdown of miR-196b-5p promoted viability and proliferation and inhibited apoptosis of HKFs (Li et al., 2022). Therefore, H19 promotes keloid progression via sponging miR-196b-5p and increasing SMAD5 expression (Figure 1).

### Homeobox (HOX) A11-AS

**FIGURE 2:**
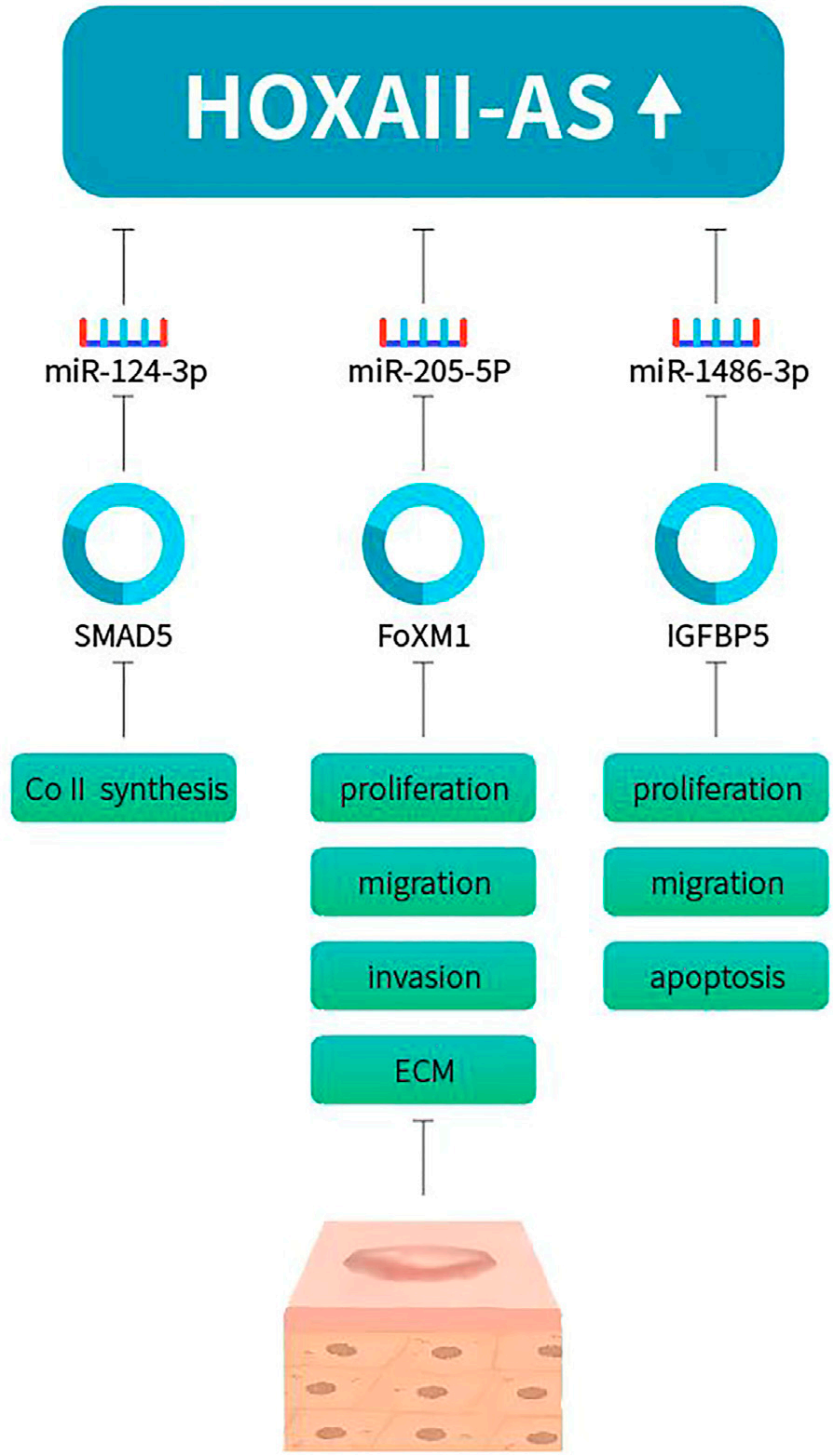
HOXA11-AS promotes keloid progression via sponging miR-124-3p/Smad5, miR-205-5p/FOXM1, miR-148b-3p/IGFBP5 axis.

LncRNA homeobox (HOX) A11 antisense, HOXA11-AS, was recently proved to promote keloid formation through inducing type I collagen (ColI) synthesis. HOXA11-AS and ColI expression levels were increased in human keloid tissues and HKFs (Jin et al., 2019). Further investigation explored the biological functions of HOXA11-AS in keloids. Knockdown of HOXA11-AS decreased cell proliferation and migration in HKFs. miR-124-3p expression level was inversely correlated with HOXA11-AS in keloid tissues and HKFs. miR-124-3p could reverse the effects of HOXA11-AS. Therefore, miR-124-3p was a target of HOXA11-AS, acting as a sponge. Previous studies had showed that Smad5 played important regulation in ColI synthesis (Wang et al., 2014). Smad5 was proved to be a target of miR-124-3p. To conclude, HOXA11-AS promote keloid formation and induced ColI synthesis through sponging miR-124-3p-mediated Smad5 signaling. Another recent study also found that HOXA11-AS-miR-205-5p-FOXM1 pathway contributes to the progression of keloid (Su et al., 2021). They showed that HOXA11-AS was upregulated in human keloid tissues and HKF. MiR-205-5p expression was inversely correlated with HOXA11-AS expression. Knockdown of HOXA11-AS hindered cell proliferation, migration, invasion, ECM deposition, and glycolysis but increased cell apoptosis of HKFs. In addition, miR-205-5p was targeted by HOXA11-AS and could reverse the effects of HOXA11-AS on keloid formation. Forkhead box M1 (FOXM1) was a target of miR-205-5p, and HOXA11-AS regulated the expression of FOXM1 by adsorbing miR-205-5p. FOXM1 overexpression abolished the role of miR-205-5p enrichment. Wang et al. proved that HOXA11-AS promoted the keloid formation by targeting miR-148b-3p/IGFBP5 axis (Wang and Shen, 2021). HOXA11-AS and IGFBP5 expression was increased while miR-148b-3p expression was reduced in keloid and HKFs. Downregulation of HOXA11-AS inhibited cell proliferation, migration and promoted apoptosis in HKFs. miR-148b-3p was a sponging target by HOXA11-AS, which abrogate the inhibition on IGFBP5, thus increasing HKFs proliferation, migration and decreasing apoptosis (Figure 2). Zhou et al. showed that HOXA11-AS was upregulated in keloid tissues and HKFs. HOXA11-AS promote keloid formation through regulating the miR-188–5p/VEGFA axis to (Zhou et al., 2022).

### CACNA1G-AS1, CAS1

LncRNA Calcium voltage-gated channel subunit alpha1 G antisense RNA 1 (CACNA1G-AS1, CAS1) is the antisense RNA of CACNA1G, mRNA of the T-type channel protein Cav3.1 (Fukunaga, 2015). CAS1 expression was higher in keloid tissue than in normal skin. CAS1 promoted cell migration in human keloid fibroblasts (HKFs). In addition, CAS1 might promote calcium channel protein and type I collagen expression (Li et al., 2018). Another study also showed that CAS1 expression was increased in keloid tissues and keloid fibroblasts. CAS1 increased HKFs proliferation and invasion and decreased apoptosis (Zhao et al., 2020b). MiR-205 was significantly downregulated in keloid tissues and HKFs, negatively regulated by CAS1. MiR-205 was a target of CAS1. Inhibited expression of miR-205 promoted HKFs proliferation and invasion and decreased apoptosis. Collectively, CAS1 increased proliferation and invasion and decreased apoptosis in HKFs through targeting miR-205.

### LINC01116

Accumulating reports demonstrated that long non-coding RNA LINC01116 was associated with the development of various malignancies (Fang et al., 2018). A recent study showed that LINC01116 promoted keloid formation through regulating miR-203/SMAD5 axis (Yuan et al., 2020). LINC01116 expression was upregulated in keloid tissues and HKFs. Knockdown of LINC01116 suppressed cell proliferation, invasion migration, and ECM production while induced apoptosis in keloid fibroblast. MiR-203 expression was inversely correlated with LINC01116 and miR-203 could directly bind to LINC01116. In summary, LINC01116 may provide a novel perspective of therapeutic target of keloid. Another study showed that LINC01116 regulates proliferation, migration, and apoptosis of HKFs by the TGF-β1/SMAD3 signaling via targeting miR-3141 (Wu et al., 2021). LINC01116 expression was increased and miR-3141 expression was reduced in human keloid tissues and fibroblasts. Knockdown of LINC01116 inhibited HKFs proliferation, migration, and increased apoptosis by directly binding to miR-3141. Moreover, knockdown of LINC01116 suppressed the subcutaneous keloid growth *in vivo*. TGF-β1 was proved to be a direct and functional target of miR-3141.

### Long non-coding RNAs-activated by TGF-β

LncRNA-activated by TGF-β (lncRNA-ATB) has been proved to promote tumor cell invasion and metastasis, exhibiting oncogenic functions in several cancers (Yuan et al., 2014; Yue et al., 2016). LncRNA-ATB expression was upregualted in keloid tissue and HKFs. Knockdown of lncRNA-ATB upregulated the expression of autocrine secretion of TGF-β and ZNF217, but increased the expression of miR-200c in KFs. ZNF217 was a transcriptional activator of TGF-β and could increase epithelial-mesenchymal transition (EMT) in breast cancer (Bai et al., 2014). To conclude, lncRNA-ATB/miR-200c/ZNF217/TGF-β2 signaling axis played crucial roles in the initiation and progression of keloids (Zhu et al., 2016).

### Long non-coding RNA AC073257.2

The lncRNA AC073257.2 and its upstream target gene Gli2 were both upregulated in keloid. LncRNA AC073257.2 and HNF1A-AS1 might regulate keloid cell growth and proliferation by its target gene GLI2 and HNF1A respectively. The differentially expressed Hh signaling pathway-related lncRNAs and mRNAs may help to uncover the pathogenesis of keloid (Huang et al., 2018).

### LINC00937

LINC00937 suppresses HKFs proliferation and ECM deposition by targeting the miR-28-5p/MC1R axis (Wan et al., 2021). LINC00937 and MC1R expression were reduced, while miR-28-5p expression was upregulated in keloid tissues, as well as in HKFs. LINC00937 overexpression could repress the extracellular matrix (ECM) deposition and cell proliferation and promote MC1R expression in HKFs. Furthermore, LINC00937 increased MC1R expression by sponging miR-28-5p. Overall, LINC00937 inhibited the ECM deposition and HKFs proliferation by sponging miR-28-5p and promoting MC1R expression.

### Wingless type signaling pathways

Wingless type (Wnt) signaling pathway was demonstrated to play a role in the pathogenesis of wound healing (Igota et al., 2013; Bastakoty and Young, 2016). Using a pathway-focused lncRNA microarray, Sun and his colleagues identified a total of 69 Wnt-related lncRNAs aberrantly expressed in keloids. A stepwise biomathematics and intracellular qPCR validation finally identified four skin-related lncRNAs, including CACNA1G-AS1, HOXA11-AS, LINC00312 and RP11-91I11.1, as biomarkers involved in Wnt-gene regulation in keloids. In-depth exploration on these four lncRNAs involved in Wnt-network contributes to the identification of the novel target in keloids (Sun et al., 2017).

### Hedgehog signaling pathway

**FIGURE 3:**
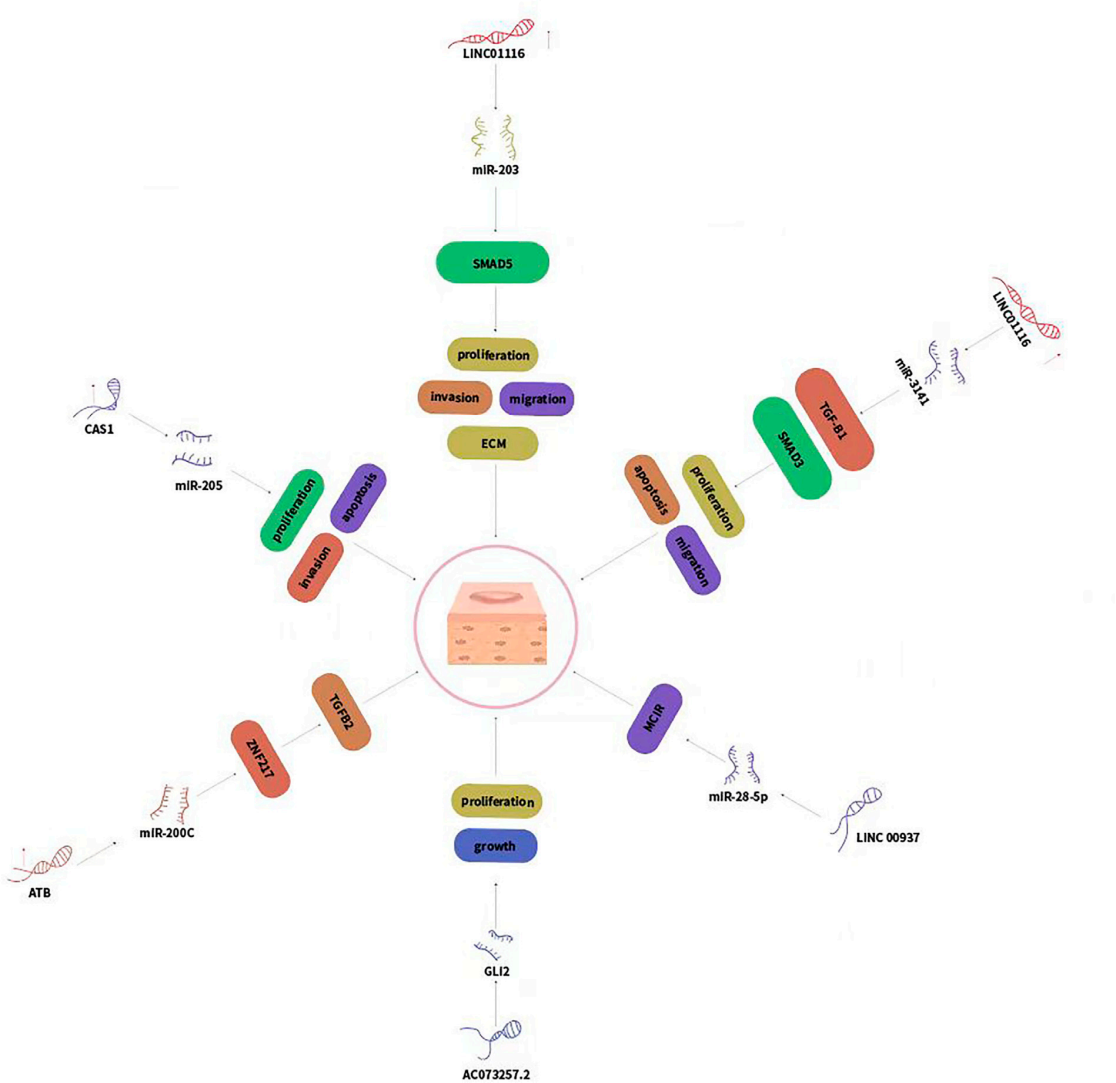
LncRNAs regulated genes expression via sponging miRNAs and played crucial roles in keloid development.

Hedgehog (Hh) signaling pathway plays important roles in several biological and pathological processes, especially in malignant tumors (Skoda et al., 2018). Huang et al. identified that 33 mRNAs and 30 lncRNAs relating to the Hh pathway were differentially expressed in keloid tissue compared with the adjacent normal skin epidermis. The upregulated mRNAs participated in cell growth, proliferation, and tissue repair while the downregulated mRNAs were participated in cell apoptosis (Figure 3).

## Summary

In this review, we summarized the published research data about the role of lncRNAs in the pathogenesis of pathological scars, including keloid and HS. In particular, we discussed the regulatory relationships between lncRNAs and miRNAs in the progression of keloid. These researches strongly support the potential role of lncRNAs keloid and HS. Some lncRNAs were upregulated and they promoted skin fibrosis while other lncRNAs were downregulated and suppressed skin fibrosis. LncRNAs regulate key processes involved in keloid and HS, including fibroblast proliferation, ECM deposition, Wnt signaling, Hh signaling and TGF-βsignaling. However, the knowledge about lncRNA-miRNA crosstalk remains largely unexplored in keloid. Investigating the expression and function of lncRNAs will help to increase our understanding about the molecular mechanisms of pathological scars. Recent studies have identified circular RNAs (circRNAs) as important regulators in human keloid (Jiao et al., 2022, 4179, 00001; Yuan et al., 2022). LncRNAs and circRNAs might serve as potential novel biomarkers for diagnosis, prognosis, and treatment for keloid. More investigation are required.
